# Single Cell Analysis Facilitates Staging of *Blimp1*-Dependent Primordial Germ Cells Derived from Mouse Embryonic Stem Cells

**DOI:** 10.1371/journal.pone.0028960

**Published:** 2011-12-15

**Authors:** John J. Vincent, Ziwei Li, Serena A. Lee, Xian Liu, Marisabel O. Etter, Silvia V. Diaz-Perez, Sara K. Taylor, Sofia Gkountela, Anne G. Lindgren, Amander T. Clark

**Affiliations:** 1 Department of Molecular Cell and Developmental Biology, University of California Los Angeles, Los Angeles, California, United States of America; 2 Eli and Edythe Broad Center of Regenerative Medicine and Stem Cell Research, University of California Los Angeles, Los Angeles, California, United States of America; 3 Molecular Biology Institute, University of California Los Angeles, Los Angeles, California, United States of America; 4 Jonsson Comprehensive Cancer Center, University of California Los Angeles, Los Angeles, California, United States of America; University of Southern California, United States of America

## Abstract

The cell intrinsic programming that regulates mammalian primordial germ cell (PGC) development in the pre-gonadal stage is challenging to investigate. To overcome this we created a transgene-free method for generating PGCs *in vitro* (iPGCs) from mouse embryonic stem cells (ESCs). Using labeling for SSEA1 and cKit, two cell surface molecules used previously to isolate presumptive iPGCs, we show that not all SSEA1+/cKit+ double positive cells exhibit a PGC identity. Instead, we determined that selecting for cKit^bright^ cells within the SSEA1+ fraction significantly enriches for the putative iPGC population. Single cell analysis comparing SSEA1+/cKit^bright^ iPGCs to ESCs and embryonic PGCs demonstrates that 97% of single iPGCs co-express PGC signature genes *Blimp1, Stella, Dnd1, Prdm14* and *Dazl* at similar levels to e9.5–10.5 PGCs, whereas 90% of single mouse ESC do not co-express PGC signature genes. For the 10% of ESCs that co-express PGC signature genes, the levels are significantly lower than iPGCs. Microarray analysis shows that iPGCs are transcriptionally distinct from ESCs and repress gene ontology groups associated with mesoderm and heart development. At the level of chromatin, iPGCs contain 5-methyl cytosine bases in their DNA at imprinted and non-imprinted loci, and are enriched in histone H3 lysine 27 trimethylation, yet do not have detectable levels of Mvh protein, consistent with a Blimp1-positive pre-gonadal PGC identity. In order to determine whether iPGC formation is dependent upon Blimp1, we generated *Blimp1* null ESCs and found that loss of Blimp1 significantly depletes SSEA1/cKit^bright^ iPGCs. Taken together, the generation of *Blimp1*-positive iPGCs from ESCs constitutes a robust model for examining cell-intrinsic regulation of PGCs during the *Blimp1*-positive stage of development.

## Introduction

The molecular events that regulate cell fate decisions in post-implantation mammalian embryonic development are largely uncharacterized due to the challenge in identifying and isolating small populations of specific precursor cells that are developmentally transient in the early embryo. In particular, precursors of the germ cell lineage are initially set aside as four to six cells in the murine embryonic epiblast, which proliferate and migrate through the primitive streak to generate the initial founder primordial germ cell (PGC) pool of approximately forty cells at the base of the allantois at embryonic (e) day e7.5 [1], [2]. The PGCs migrate out of the allantois and into the embryonic hindgut endoderm at e8.0–8.5 where they continue to proliferate and begin to accumulate nuclear histone H3 lysine 27 trimethylation (H3K27m3) [3]. By e10.5–e11.0 a single embryo has approximately 1,000–2,000 PGCs, which exit the hindgut and begin colonization of the indifferent gonad and express Mvh protein [3], [4], [5].

The transcription factors that specify and sustain PGC identity prior to gonadal colonization are not well understood. One of the most characterized regulators of PGC fate is the transcriptional repressor *B-lymphocyte induced maturation protein 1 (Blimp1)*, the transcriptional product of the *PRD1-BF1 and RIZ (PR) domain 1 (Prdm1)* gene. Blimp1 expression is detected in epiblast-derived PGCs and persists until e11.5, when PGCs have colonized the gonad [2], [6]. Loss of one *Prdm1* allele significantly reduces PGC numbers in the allantois, with the loss of both causing almost a complete loss of PGCs [2]. The major direct target of Blimp1 in PGCs is hypothesized to be *Hoxb1* [7]. However, direct binding of Blimp1 at the *Hoxb1* locus in PGCs has not been demonstrated.

The mechanism by which Blimp1 mediates gene repression is hypothesized to involve recruitment of the chromatin-remodeling enzyme Protein arginine methyltransferase 5 (Prmt5) to chromatin [6]. However, genome-wide analysis of PGC chromatin is currently not feasible due to the challenge in performing chromatin immunoprecipitation on small cell numbers, necessitating the development of a scalable model to accurately capture the Blimp1-positive phase of PGC development.

The differentiation of pluripotent stem cells, including embryonic stem cells (ESCs), has emerged as a novel technology for generating sufficient numbers of embryonic progenitors at-scale to evaluate embryonic lineage development. A number of methods for identifying *in vitro* PGCs (iPGCs) have been described that mostly involve use of integrated fluorescent reporters, including *Oct4-delta-PE-Gfp* [8], [9], [10], [11], [12], *Stella*-*Gfp* [13], [14], [15], *Dazl-Gfp* [16] and *Mvh-LacZ/Rfp* transgenes [10], [17], [18]. A small number of studies have used Stage Specific Embryonic Antigen 1 (SSEA1) to enrich for germ cells [19], [20], but the identity of PGCs from ESCs within the SSEA1+ fraction has not been interrogated at a single cell level. Furthermore, the majority of PGC differentiation studies have been designed to characterize the post-colonized Blimp1-negative PGC. Therefore, the goal of the current study is to generate a robust ESC differentiation system to acquire PGCs in the Blimp1-positive stage of development for future in-depth analysis of the pre-gonadal stage.

## Results

### cKit^bright^ refines an Oct4+/SSEA1+ iPGC population in embryoid bodies

**Figure 1:**
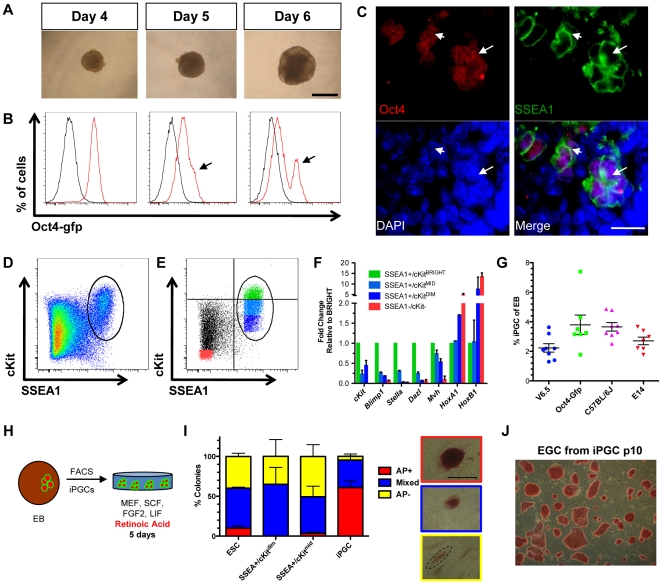
Transgene-free method for isolating iPGCs from embryoid bodies. **A:** V6.5 embryoid bodies in hanging drops at days 4, 5, and 6 of differentiation. Scale bar = 500 microns. **B:** Oct4-Gfp expression (red) relative to V6.5 EBs (black) at days 4, 5 and 6 of differentiation. Arrows indicate shoulder of Oct4-Gfp^bright^ cells at day 5 and an Oct4-Gfp^bright^ peak at day 6. **C:** Immunofluorescence of EBs at day 6 for Oct4 (red) and SSEA1 (green). Double positive cells localize in discreet clusters (arrow). Scale bar = 20 microns. **D:** Flow cytometry plot of V6.5 day 6 EBs stained for SSEA1 and cKit. Oval gate defines the SSEA1+/cKit+ side population. **E:** Flow plot day 6 EBs from V6.5 ESCs fractionated by expression of SSEA1 and cKit into SSEA1+/cKit^bright^ (green), SSEA1+/cKit^mid^ (light blue), SSEA1+/cKit^dim^ (dark blue), and SSEA1-/cKit- cells (red) populations. Quadrant gates are drawn to demonstrate the criteria for selecting SSEA1+/cKit^bright^ cells. The remaining cKit+ population was split into two equal fractions, mid and dim. **F:** Semi-quantitative real-time PCR from the populations isolated in E, with levels normalized to *Gapdh*. SSEA1+/cKit^bright^ cells are set at 1.0. Data is from two biological replicates each performed in technical duplicate. Error bars represent s.e.m. **G:** Percentage of live iPGCs acquired from differentiation of ESCs of different genetic backgrounds. Each line was tested at least seven independent times. **H:** Diagrammatic representation of iPGC replating assay. **I:** Quantification of alkaline phosphatase (AP) staining of colonies derived from indicated cell populations after 5 days of culture. Right, representative images of colony types. Scale bar = 500 microns. **J:** Self-renewing EGCs at passage 10 derived from RA/FGF2/LIF/SCF cultured iPGCs, followed by routine passaging in the presence of LIF only. Error bars represent s.d.

To identify pre-gonadal iPGCs with differentiation, we first used *Oct4-Gfp* ESCs [21] to generate hanging-drop embryoid bodies (EBs) containing 300 cells per drop (Figure 1A). EBs could be maintained for up to 8 days in this system (Figure S1A), but cell viability decreased rapidly after day 6 from 69% to 19% by day 8 (Figure S1B). Using flow cytometry we show that Gfp is retained in the majority of cells in the first four days of differentiation (Figure 1B), reminiscent of sustained Oct4 expression *in vivo* in both PGCs and embryonic somatic cells up to e8.5 [22], [23]. On day 5 of differentiation, we observed the emergence of a shoulder of Gfp^bright^ cells and the formation of a distinct Gfp+ peak by day 6 (Figure 1B, arrow).

To generate a transgene-free method of iPGC differentiation, we correlated expression of Oct4 protein in day 6 EBs derived from V6.5 ESCs with the cell surface marker SSEA1. In the embryo, SSEA1 is highly expressed on Blimp1-positive stage PGCs and PGC precursors derived from epiblast stem cells [24], [25]. We found that Oct4 is co-expressed with SSEA1 in small cell clusters at day 6 of differentiation by immunofluorescence (Figure 1C). Given that Oct4 and SSEA1 are also expressed by undifferentiated ESCs, we used the membrane-localized tyrosine kinase receptor cKit to assist in further defining the iPGC population within either the SSEA1+ or Oct4+ fractions. *cKit* is highly expressed by endogenous PGCs from e7.25 to e13.5 [7], [22], [26] and is not expressed by epiblast cells [22]. Indeed, flow cytometry analysis of V6.5 ESC-derived EBs at day 6 of differentiation revealed a discreet a side population of SSEA1+/cKit+ cells (Figure 1D). A side population of Oct4-Gfp+/cKit+ cells was also identified beginning at day 6 of differentiation in EBs derived from *Oct4-gfp* ESCs, and this was sustained to day 8 (Figure S1C).

To interrogate PGC identity in specific fractions of SSEA1+/cKit+ cells when the population is first identified at day 6, we used real time PCR to determine relative levels of PGC-expressed genes in discreet cKit+ fractions. These fractions include SSEA1+/cKit^bright^ (green), SSEA1+/cKit^mid^ (light blue), and SSEA1+/cKit^dim^ (dark blue), and SSEA1-/cKit- cells (red) as a negative control (Figure 1E). cKit^bright^ cells were selected based on increased signal intensity above the main population. PGC genes including *cKit*, *Blimp1*, *Stella* and *Dazl* were all enriched in the SSEA1+/cKit^bright^ fraction, with lower expression in the cKit^mid^ and cKit^dim^ fractions of SSEA1+ cells (Figure 1F). *Mvh* was also expressed in the SSEA1+/cKit^bright^ fraction, but was not specifically enriched in SSEA1+/cKit^bright^ cells relative to cKit^mid^ and cKit^dim^. Furthermore, analysis of *Mvh* levels in SSEA1+/cKit^bright^ cells at day 8 of differentiation also did not show an increase relative to day 6 (data not shown). In contrast, transcription factors expressed in somatic cells such as *Hoxa1* and *Hoxb1* were highly expressed in SSEA1-/cKit- and SSEA1+/cKit^dim^ relative to SSEA1+/cKit^bright^ cells. Together, we conclude that not every SSEA1+/cKit+ cell in EBs at day 6 of differentiation is a putative PGC, and that selecting for cKit^bight^ cells enriches for the iPGCs beginning at day 6 of differentiation and persisting until day 8. We next determined if Oct4-gfp could further sub-fractionate the putative SSEA1+/cKit^bright^ putative iPGC population, and found equal enrichment of Oct4-gfp in all SSEA1+ cells regardless of cKit intensity (Figure S1D). Therefore the use of the Oct4-Gfp reporter together with SSEA1 and cKit does not further refine the isolation of putative iPGCs, but instead shows that Oct4-Gfp and SSEA1 report the same population when used with cKit.

We next evaluated the yield of SSEA1+/cKit^bright^ or Oct4-gfp+/cKit^bright^ cells (called iPGCs) isolated at day 6 of differentiation (Figure 1G). We determined that 1–4% of total live EB cells exhibited an iPGC surface signature, and there was no statistical difference between genetic backgrounds (Figure 1G). Furthermore, gene expression profiling of Oct4-gfp+/cKit^bright^ cells from the *Oct4-gfp* line and SSEA1+/cKit^bright^ cells from J1 EBs at day 6 revealed enriched expression of *cKit, Stella, Blimp1, Dazl* and *Mvh* relative to the SSEA1-/cKit- somatic cell controls (Figure S1E). Conversely, somatic gene expression as documented by *Hoxa1* and *Hoxb1* were not enriched in the putative iPGCs relative to somatic cells (Figure S1F).

To test identity of SSEA1+/cKit^bright^ cells derived from V6.5 ESCs we sorted putative iPGCs and cultured them on mouse embryonic fibroblasts (MEFs) supplemented with basic Fibroblast Growth Factor 2 (FGF2), Stem Cell Factor (SCF), Leukemia Inhibitory Factor (LIF) and retinoic acid (RA), a driver of PGC proliferation (Figure 1H). This assay has been used previously to confirm PGC identity relative to undifferentiated ESCs, which respond to RA by undergoing differentiation and becoming alkaline phosphatase (AP) negative [15], [19], [20]. AP+ colony forming ability is almost exclusively associated with SSEA1+/cKit^bright^ population when compared to sorted undifferentiated ESCs, SSEA1+/cKit^mid^ or SSEA1+/cKit^dim^ cells plated at equivalent numbers (Figure 1I). The three non-iPGC populations generate mostly mixed colonies or AP negative colonies. Withdrawal of RA, FGF2 and SCF while retaining LIF in the media of RA-treated SSEA1+/cKit^bright^ sorted cells results in the formation of self-renewing pluripotent embryonic germ cells (EGCs), which could be maintained for at least 10 passages (Figure 1J).

### Day 6 iPGCs have a pre-gonadal, pre-reprogrammed PGC identity

**Figure 2:**
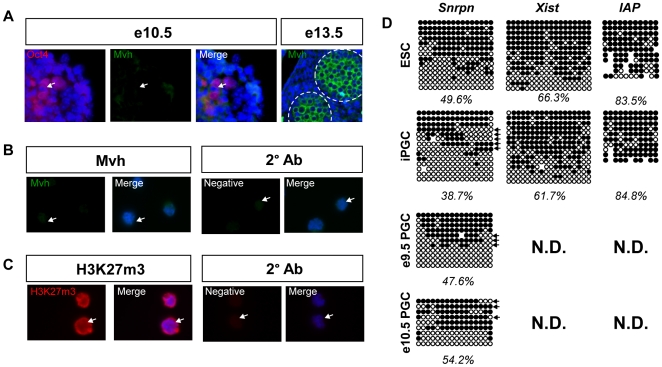
iPGCs have characteristics of pre-gonadal, pre-reprogrammed *in vivo* PGCs. **A:** Immunofluorescence of pre-gonadal e10.5 PGCs stained for Oct4 (red) and Mvh (green). e13.5 male gonadal PGCs were stained as a positive control. Dotted circles mark the testis cords. **B:** Sorted SSEA1+/cKit^bright^ iPGCs stained for Mvh (green, left) or secondary antibody alone (right). **C:** iPGCs stained for H3K27m3 (red, left), and secondary antibody alone (right). Arrows point to individual iPGCs. **D:** Bisulfite sequencing of ESCs, iPGCs, and endogenous e9.5 PGCs for *Snrpn*, the *Xist* promoter, and *IAP*. Circles represent individual CG dinucleotides, black = methylated and white = unmethylated cytosines. Arrows indicate individual alleles that display characteristic demethylation. N.D. = not determined.

Day 6 EB-derived SSEA1+/cKit^bright^ or Oct4-Gfp+/cKit^bright^ iPGCs consistently express *Dazl* and *Mvh* RNA in addition to *Blimp1* (Figure 1F & Figure S1E). Therefore we could hypothesize that putative iPGCs correspond to newly colonized PGCs that have expressed Mvh protein and have potentially undergone whole genome reprogramming. To address this, we performed immunofluorescence for Mvh, which is first detectable in gonadal PGCs at e11.5 [27], [28], [29]. We also evaluated DNA demethylation at imprinted and non-imprinted genes, which is erased by e12.5 [30], [31]. Immunofluorescence analysis of e10.5 embryos with antibodies against Mvh and Oct4 confirms that e10.5 Oct4-positive PGCs are negative for Mvh protein, whereas gonadal-stage PGCs are Mvh positive (Figure 2A). Analysis of SSEA1+/cKit^bright^ sorted iPGCs derived from V6.5 ESCs reveals that Mvh protein is not detectable above background (Figure 2B). We also tested J1 ESC-derived iPGCs and were unable to detect Mvh protein similar to V6.5 iPGCs (data not shown). Furthermore we evaluated H3K27m3 in SSEA1+/cKit^bright^ iPGCs, a histone modification that is depleted from the PGC genome from e11.5–e12.5 [4]. We found a high nuclear content of H3K27me3 in iPGCs (Figure 2C). Together this data suggests that the SSEA1+/cKit^bright^ iPGCs are pre-reprogrammed and younger than e11.5.

To further confirm a pre-reprogrammed identity, we next evaluated the methylation status of an imprinted gene (*Snrpn*) and two non-imprinted loci, *Xist* and *Intracisternal A Particle 1 (IAP)*, by bisulfite sequencing (Figure 2D). Analysis of undifferentiated ESCs shows that the differentially methylated region (DMR) of *Snrpn* is 49.6% methylated, the *Xist* promoter is 66.3% methylated, and *IAP* is 83.5% methylated. In the putative iPGCs, methylation at the *Snrpn* DMR is modestly reduced to 38.7%, while *Xist* and *IAP* methylation levels are the same as ESCs. To determine if the DMR of *Snrpn* also exhibits partial demethylation in endogenous PGCs, we performed bisulfite sequencing of sorted PGCs from e9.5 and e10.5 *Oct4-gfp* embryos. Methylation at the *Snrpn* DMR in e9.5 and e10.5 PGCs from the embryo were still present (47.6% and 54.2% respectively) consistent with previously published findings [31]. Furthermore, we observed evidence of demethylation at the 5′ and 3′ ends of three clones in endogenous PGCs at e9.5 and two sequences at e10.5 similar to what was observed in iPGCs (Figure 2D, arrows). Taken together, using real time PCR, immunofluorescence and bisulfite sequencing, our data strongly argue that the Blimp1-positive PGCs isolated from EBs at day 6 of differentiation correspond to a pre-e11.5 stage germ cell *in vitro*.

### Germ line signature genes including *Blimp1* are co-expressed in single SSEA1+/cKit^bright^ cells

**Figure 3:**
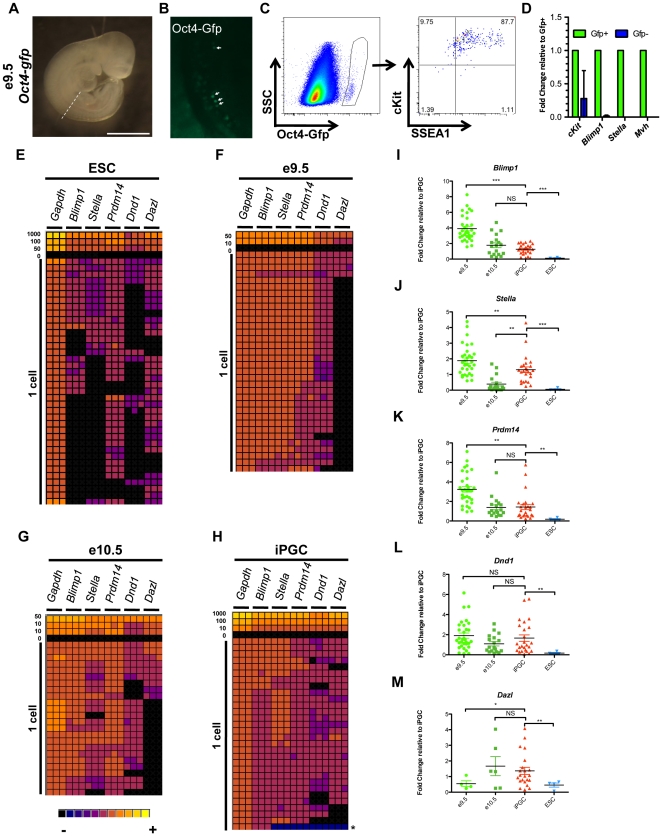
Developmental staging of pre-gonadal iPGCs at single cell resolution. **A:** Bright field image of representative e9.5 *Oct4-gfp* embryo. Dotted line indicates where the embryo was bisected at somite 13 for FACS. Scale bar = 1 mm. **B:** Whole mount confocal microscopy of live embryos with migratory Oct4-gfp+ PGCs within the hindgut (arrows). **C:** Flow cytometry of the bisected lower half of e9.5 *Oct4-gfp* embryos. Oct4-gfp+ PGCs (circled gate) are also positive for SSEA1 and cKit. **D:** Real-time RT-PCR of Gfp+ and Gfp- cells. Error bar denotes s.d. **E–H:** Gene expression analysis at single cell resolution for ESCs (E), e9.5 PGCs (F), e10.5 PGCs (G), and iPGCs (H) represented as a heat map of CT values with expression ranging from not detected (black) to high (yellow). A cell titration was performed as a control to ensure linear amplification of each primer set. Each cell was evaluated for the expression of each gene in technical triplicate. **I–M:** Semi-quantitative analysis of single cell real time PCR in E–H of cells that co-express *Blimp1*, *Stella*, *Prdm14*, and *Dnd1* expressed relative to the average delta CT expression level for each gene in single iPGCs. *p<1e-03, **p<1 e-04, ***p<1 e-06, NS = not significant.

To determine whether the relative levels of PGC signature genes in SSEA1+/cKit^bright^ cells are comparable to the levels found in PGCs sorted from the embryo prior to e11.5, we sorted Gfp+ cells from *Oct4-gfp* embryos at 9.5 and e10.5 (Figure 3A–C, shown is e9.5). A distinct Gfp+ population was detected from e9.5 to at least e13.5 (Figure 3C and data not shown). Sorted Oct4-gfp+ PGCs from the embryo are SSEA1+ and exhibit bright cKit+ staining (Figure 3C). We confirmed that the Gfp+ cells are PGCs due to enriched expression of *cKit*, *Blimp1*, *Stella*, and *Mvh* relative to the Gfp- somatic cells at a population level by real time PCR (Figure 3D). Detection of *cKit* and *Blimp1* RNA in the Gfp- population was not unexpected as these genes are also expressed in endothelial and hematopoietic cells during early embryogenesis [32], [33].

We next evaluated the transcriptional identity of undifferentiated ESCs, iPGCs and embryonic PGCs at e9.5 and e10.5 at a single cell level by examining expression of five signature PGC markers (*Blimp1, Stella, Prdm14, Dnd1* and *Dazl*) using the BioMark Fluidigm Real Time PCR platform (Figure 3E–H). We evaluated 38 single undifferentiated ESCs (Figure 3E), 34 single embryonic Oct4-Gfp+ PGCs at e9.5 (Figure 3F), 24 single Oct4-Gfp+ PGCs at e10.5 (Figure 3G), and 30 iPGCs from day 6 EBs (Figure 3H). In undifferentiated ESCs, 17 of the 38 cells (44%) expressed *Blimp1*. Of the 17 *Blimp1*+ cells, 6 did not express *Stella* and 12 did not express *Dnd1*. In contrast to ESCs where less than 50% of cells expressed *Blimp1*, 100% of e9.5 and e10.5 PGCs from the embryo and iPGCs expressed *Blimp1* (Figure 3F–H). Heat maps of the single cell analysis indicate that e9.5 PGCs are relatively homogeneous when comparing individual cells to each other for each gene, whereas at e10.5 and in iPGCs expression levels between individual cells is more heterogeneous (Figure 3G,H). Critically, only one cell in the iPGC cohort was not a germ cell (Figure 3H, asterisk).

We next examined expression levels of each gene for all cells that co-expressed *Blimp1*, *Stella*, *Dnd1*, and *Prdm14* relative to levels in SSEA1+/cKit^bright^ iPGCs. We first compared ESCs to iPGCs and found that of the 4 *Blimp1+* ESCs that co-expressed *Stella*, *Prdm14* and *Dnd1* (10.5%), the transcript levels were significantly lower than those in iPGCs (Figure 3I–L). However, comparison of iPGCs to embryonic e10.5 PGCs revealed no significant difference with regard to *Blimp1, Prdm14*, and *Dnd1* expression levels (Figure 3I,K–L). In single cells that also co-expressed *Dazl*, ESCs displayed significantly diminished *Dazl* levels, but no significant difference was found between iPGCs and e10.5 endogenous PGCs (Figure 3M). *Stella* levels were statistically different between all groups, with SSEA1+/cKit^bright^ iPGCs on average expressing intermediate levels between e9.5 and e10.5 embryonic PGCs (Figure 3J). We propose that the intermediate levels of *Stella* in iPGCs between e9.5 and e10.5 PGCs may indicate that iPGCs are developmentally equivalent to a period of germ cell differentiation between e9.5 and e10.5.

### Gene expression profiling by microarray reveals iPGCs repress a mesoderm transcriptional program and identifies a novel marker of *in vitro* PGC formation from ESCs

**Figure 4:**
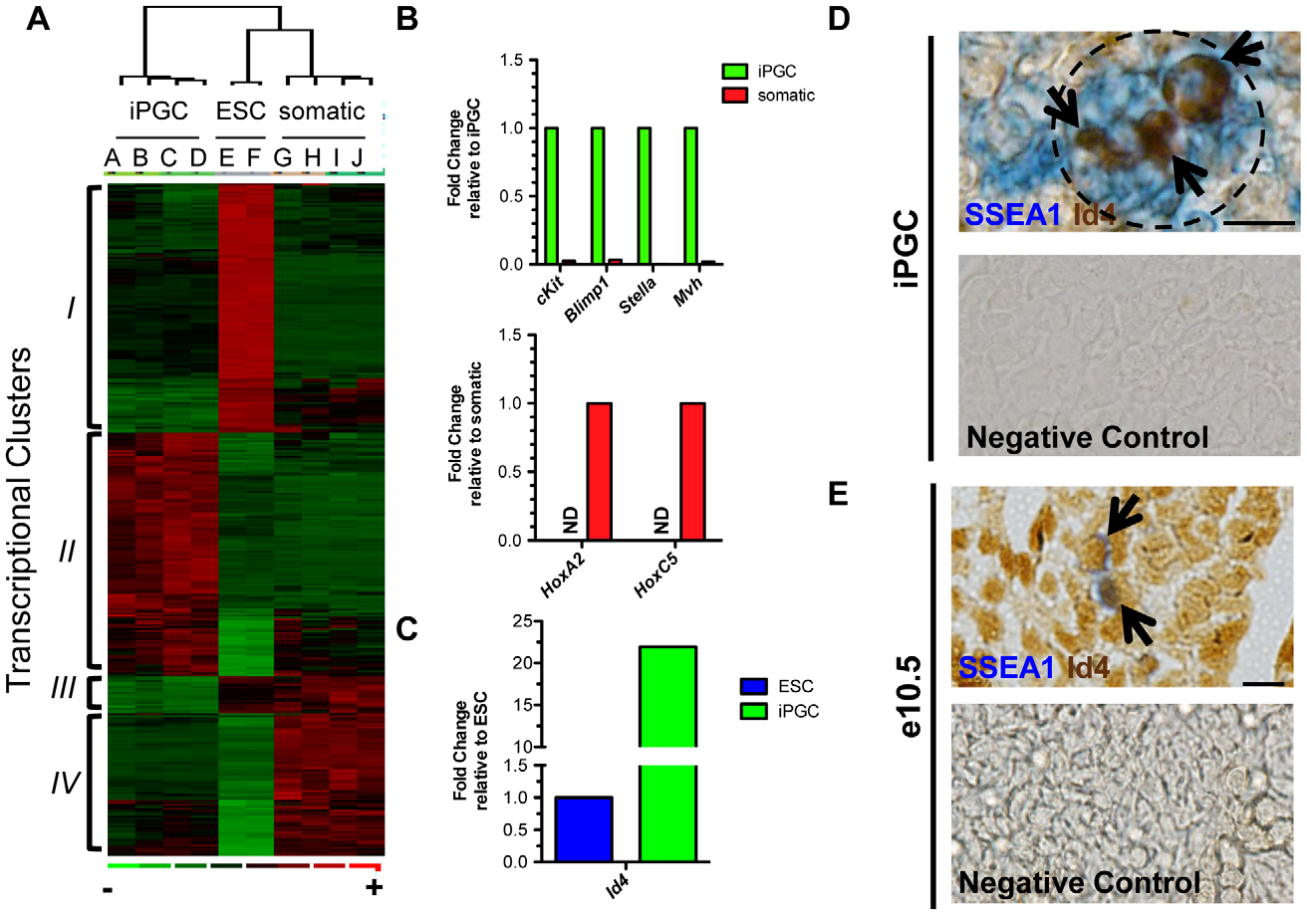
Transcriptional profiling demonstrates a PGC program and identifies novel markers for *bona fide* iPGCs from ESCs. **A:** Microarray analysis comparing iPGCs (A,B,C,D), V6.5 ESCs (E and F), and the somatic cells of the EB (G through J). Genes differentially expressed by threefold between undifferentiated ESCs and iPGCs are shown (p<0.01). Red indicates significant up-regulation, green repression and black no change. **B:** Semi-quantitative RT-PCR validation of microarray in Oct4-Gfp/cKit^bright^ iPGCs versus Gfp-/cKit- somatic cells. ND = no transcript detected. **C:** Real time RT-PCR of *Id4* in V6.5 ESCs (set to 1.0) compared to iPGCs. **D:** Immunohistochemistry of day 6 V6.5 EBs for SSEA1 (blue) and Id4 (brown, arrows). Dotted black line denotes SSEA1+ cluster within the EB. Scale bar = 10 microns. **E:** Immunohistochemistry of e10.5 embryos. Id4 is expressed in SSEA1+ PGCs (arrows) and somatic cells. Scale bar = 20 microns. Negative controls were performed with secondary antibodies alone.

Although analysis of five critical PGC-expressed genes at a single cell level was informative for ensuring that >96% of SSEA1+/cKit^bright^ iPGCs have a *Blimp1+* PGC identity, our next goal was to obtain a more comprehensive transcriptional portrait of iPGCs derived from day 6 EBs by performing microarray analysis using Affymetrix Mouse Genome chips followed by D-Chip analysis and examine expression of ESC-expressed genes and somatic genes (Figure 4A,B). We profiled the SSEA1+/cKit^bright^ fraction from V6.5 EBs at day 6 of differentiation (Samples A and B), Oct4-Gfp+/cKit^bright^ cells from day 6 *Oct4-gfp* EBs (Samples C and D), undifferentiated V6.5 SSEA1+/cKit+ ESCs (Samples E and F), and SSEA1-/cKit- and Oct4-/cKit- day 6 EB cells (Samples G–J).

Cluster analysis of genes that are differentially expressed at greater than three-fold between undifferentiated ESCs and iPGCs (348 genes, p<0.01) generated four major transcriptional clusters (Figure 4A & Table S1). Gene ontology (GO) analysis of Cluster *I* (enriched in ESCs but not iPGCs or somatic cells) identified genes associated with transcription factor activity and DNA binding. Cluster *II* (enriched in iPGCs but not ESCs or somatic cells) revealed enrichment in genes associated with hydrolyase activity, cytoplasmic proteins and MAPK signaling pathways. Genes in Cluster *III* (enriched in ESCs and somatic cells but not iPGCs) were associated with GO terms for stress fibers and actin filament bundle genes. Finally, GO analysis of Cluster *IV* (repressed in iPGCs and ESCs but not somatic cells) revealed genes associated with mesoderm formation including heart and blood development, and morphogenesis. Together, these data suggest that iPGCs repress genes associated with mesoderm differentiation, similar to what has been proposed for endogenous PGC formation through the activity of *Blimp1* [2], [7]. Expression of candidate PGC genes from the microarray was validated by real time RT-PCR from Oct4-Gfp*+*/cKit^bright^ iPGCs (Figure 4B). Similarly, expression of additional somatic genes (*Hoxa2* and *Hoxc5*) which were not evaluated earlier (Figure S1F) revealed undetectable expression in iPGCs, whereas somatic cells were positive.

Next, we compared our microarray data between iPGCs and undifferentiated ESCs to identify a marker that could distinguish between these two cell types. We identified *Inhibitor of DNA binding 4 (Id4)* as being significantly higher in iPGCs relative to ESCs. We confirmed the microarray data showing significant enrichment of *Id4* RNA in independently collected iPGC samples relative to undifferentiated ESCs (Figure 4C). To determine if Id4 protein is expressed in iPGCs, we performed immunohistochemistry of day 6 EBs with SSEA1 and Id4, and identified Id4 positive cells within the clusters of SSEA1+ cells (Figure 4D). Likewise, immunohistochemistry of e10.5 embryos shows that Id4 protein is expressed in SSEA1+ PGCs (Figure 4E, arrow). However, Id4 was also expressed in the surrounding embryonic somatic cells. Taken together, Id4 is a new marker for distinguishing iPGCs from undifferentiated ESCs, but does not distinguish PGCs from somatic cells of the embryo.

### *Blimp1* is specifically required for iPGC differentiation from EBs

**Figure 5:**
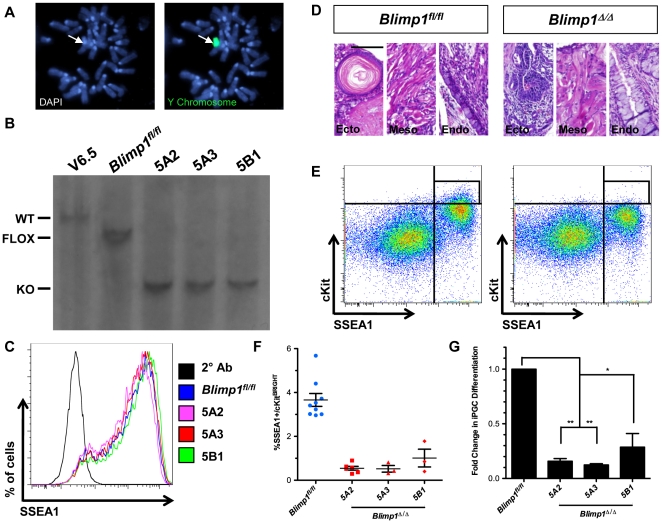
*Blimp1* is required for the differentiation of iPGCs from ESCs. **A:** DNA-FISH for the Y chromosome in *Blimp1^fl/fl^* ESCs. **B:** Southern blot for detection of wild type (WT), flox, and knock-out (KO) alleles of *Blimp1*. **C:** Flow cytometry for SSEA1 on undifferentiated ESCs. **D:** Representative histological sections from *Blimp1^fl/fl^* and *Blimp1^Δ/Δ^* teratomas. All lines were capable of differentiation to ectoderm (Ecto), mesoderm (Meso) and endoderm (Endo). Scale bar = 100 microns. **E:** Representative paired EB differentiations of *Blimp1^fl/fl^* and *Blimp1^Δ/Δ^* ESCs. Quadrant gates indicate criteria for gating SSEA1+/cKit^bright^ iPGCs, which are contained within the rectangular gate (black lines). **F:** Percentage iPGC yield in the control *Blimp1^fl/fl^* line and *Blimp1^Δ/Δ^* sub-lines. Error bars represent s.e.m. **G:** Quantification of data from F, expressed as a percent of the *Blimp1^fl/fl^* iPGC yield from each paired experiment. Error bars represent the standard error of the mean. *p<0.05, ** p<1×10^−7^.

Dosage of *Blimp1* is essential for the specification of PGCs *in vivo* [2], [32]. To determine if the emergence of SSEA1+/cKit^bright^ PGCs *in vitro* is similarly dependent upon *Blimp1* expression, we derived *Blimp1^fl/fl^* ESCs from e3.5 blastocysts. We performed Y chromosome FISH to identify a male line (Figure 5A), and generated three independent *Blimp1* knockout sub-lines (*Blimp1^Δ/Δ^*) via transfection of Cre recombinase fused to Gfp followed by re-plating of Gfp+ cells at limiting dilutions. Clones were screened by Southern blot to verify *Blimp1* deletion (Figure 5B). To compare overall self-renewal and pluripotency in *Blimp1^Δ/Δ^* cells relative to the parental line, we performed flow cytometry for SSEA1 under self-renewing conditions in the presence of LIF (Figure 5C), and teratoma analysis by injection of undifferentiated ESCs into the testicles of SCID mice (Figure 5D). In both assays, all *Blimp1^Δ/Δ^* lines were indistinguishable from parental *Blimp1^fl/fl^* cells, indicating that loss of *Blimp1* does not cause gross defects in overall ESC self-renewal or differentiation.

Finally, to evaluate *in vitro* PGC formation, we performed paired differentiation experiments with *Blimp1^fl/fl^* and *Blimp1^Δ/Δ^* lines and evaluated iPGC differentiation by flow cytometry (Figure 5E). Quantification of SSEA1+/cKit^bright^ cells revealed that iPGCs constitute approximately 3–4% of the live cell EB population in the parental *Blimp1^fl/fl^* line at day 6 (Figure 5F). In contrast, all *Blimp1^Δ/Δ^* sub-lines displayed between a 70–90% decrease in SSEA1+/cKit^bright^ iPGCs, with the average percentage constituting less than 1% of the EB in all three sub-lines examined (Figure 5F,G). Functionally, this demonstrates that sorting for SSEA1+/cKit^bright^ iPGCs captures a *Blimp1-*dependent PGC population *in vitro*, whereas generation of SSEA1+/cKit^mid^ and SSEA1+/cKit^dim^ fractions of EBs do not exhibit the same reliance on *Blimp1* as *in vivo* PGCs.

## Discussion

Emerging cell populations in the early embryo are challenging to investigate. Therefore, we used mouse ESCs from multiple genetic backgrounds to differentiate transgene-free, pre-gonadal stage PGCs where 100% of the single iPGCs express *Blimp1 in vitro*. Here we show that sorting for the cKit^bright^ fraction of SSEA1+ cells at day 6 of differentiation when the population is first discernable yields an iPGC population with an identity suggestive of PGCs younger than e11.5.

One of the major challenges in the ESC and PGC fields has been to distinguish early progenitor PGCs from undifferentiated ESCs due to their similar expression patterns. Indeed, e11.5 PGCs isolated from the genital ridge prior to sex determination cluster very closely to undifferentiated ESCs in 2-dimensional principle component analysis after microarray [11]. Therefore, it has been proposed that ESCs originate from a progenitor germ cell consistent with detectable expression of PGC-signature genes, including *Dazl* and *Tissue non specific alkaline phosphatase* in the undifferentiated state [34], [35]. Although our studies do not address the origin of ESCs, our data does indicate that a small nascent PGC-like population corresponding to about 10% of cells can be identified in an ESC culture in the self-renewing state, agreeing strongly with previous work which demonstrated that *Dazl* null ESCs exhibit reduced expression of PGC-signature genes [8]. However, our data also show that despite co-expression of germ cell genes in these 10% of cells, the transcript levels are significantly lower than the levels found in *bona fide* PGCs isolated form the embryo between e9.5–e10.5 as well as the iPGCs. Taken together, our data argues that the majority of undifferentiated ESCs are not PGCs, and that a single cell analysis is critical to uncouple differences between ESCs and progenitor PGCs.

**Figure 6:**
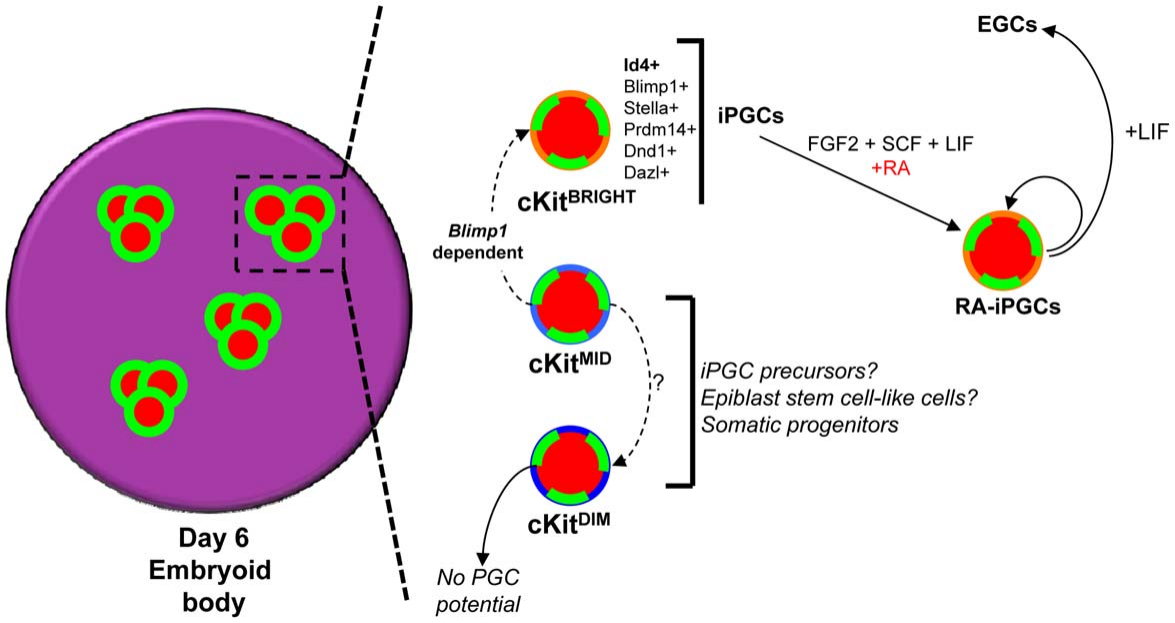
Model for iPGC emergence from SSEA1/Oct4+ clusters in EBs. In day 6 of EBs (pink circle) multiple discreet clusters of Oct4+ (red) and SSEA1+ (green) cells are identified. SSEA1+ cells within these clusters exhibit a range of cKit signal intensities identified by flow cytometry including cKit^bright^ (iPGCs), cKit^mid^ (iPGC precursors and epiblast stem cells) and cKit^dim^ (somatic lineage primed epiblast) cells (black box). Definitive iPGCs are enriched in the cKit^bright^ fraction of SSEA1+ or Oct4+ cells, and the generation of this population *in vitro* is dependent upon *Blimp1*. Using a differential colony forming assay in the presence FGF2, SCF, LIF and RA which promotes survival and proliferation of PGCs, we show that RA-iPGC potential is highest in the cKit^bright^ fraction and is absent in the cKit^dim^ subpopulation of SSEA1+ cells. Furthermore, converting RA-iPGCs to media containing LIF supports the generation of self-renewing EGCs *in vitro*.

In the current study, we identified Id4 as a new marker enriched in iPGCs relative to undifferentiated ESCs. Id4 was recently found to be a germ line marker expressed in gonocytes and spermatogonia of postnatal and adult murine gonads [36]. We extend these findings to show that Id4 is expressed during the earliest stage of germ line development, prior to gonadal colonization (Figure 4E). Interestingly, *Id4* similar to *Stella* constitutes a marker for defining PGC identity yet has no functional role in specifying PGC fate [13], [36], [37]. However, by combining Id4, SSEA1, and Oct4 expression in day 6 EBs, we propose a model for germ line formation *in vitro* that involves the generation of multiple SSEA1+/Oct4+ niches during EB formation, with Id4+ iPGCs emerging from within these niches (Figure 6). We propose that similar to PGC development in the allantois of the embryo, the tight clustering of SSEA1+/Oct4+ cells creates a microenvironment in the EB to protect the iPGCs against somatic cell differentiation signals [38]. Given that Id4+ cells constitute only a subpopulation of cells within SSEA1+ clusters, we hypothesize that the clusters are composed of a heterogeneous mixture of immature cells, including epiblast-like cells (*Stella* negative, *Blimp1* negative), PGC precursors (*Stella* negative, *Blimp1* positive) and definitive Id4-positive cKit^bright^ iPGCs (Figure 6). Whether the SSEA1+/cKit^bright^ PGCs emerge from a subpopulation of the SSEA1+/cKit^mid^ fraction of cells remains to be determined. However, our data strongly argue that iPGCs do not differentiate from SSEA1+/cKit^dim^ cells, which have no colony forming potential, and express high levels of *Hoxa1* and *Hoxb1*, indicating commitment to a somatic fate.

Although our data suggest that the iPGCs are younger than e11.5 of development due to lack of Mvh protein expression, it is conceivable that iPGCs at day 6 are more similar to e11.5 in some aspects, but have not received the appropriate cues to express Mvh protein. The signals that promote Mvh protein expression in PGCs at e11.5 are not well understood, but one study has indicated that gonadal somatic cells are involved in this process [29]. Lack of Mvh protein expression in our model suggests that the hanging drop EB system by day 6 of differentiation does not provide the necessary signals to promote developmental progression to Mvh protein-positive iPGCs. This result implies that progression of iPGCs *in vitro* may require a gonadal niche to promote differentiation to the Mvh protein-positive stage. Indeed, while this manuscript was under review, Hayashi and colleagues demonstrated that a neonatal seminiferous tubule niche was necessary to promote differentiation of ESC-derived PGCs, which this group called PGC like cells (PGCLCs), into functional post-meiotic male germ cells [39]. In these studies, PGCLCs were isolated using SSEA1 and Integrin Beta 3 and were hypothesized to be equivalent to e9.5 of development. Similar to this group, iPGCs isolated at day 6 also express significantly high levels of *Integrin Beta 3* RNA (Figure S2).

In the current study, we successfully acquired PGCs in the *Blimp1*-positive stage of development. *Blimp1* is not expressed in meiotic or post-meiotic cells and therefore our model is not useful for evaluating meiotic progression; however, we propose that this model can be used to successfully evaluate molecular events in PGC formation prior to gonadal colonization, gonadal reprogramming and sex determination. As an example of the utility of our model, one hundred male e10.5 embryos would be required to obtain approximately 100,000 PGCs via FACS, if we estimate that there are ∼1,000 PGCs per embryo at this developmental age [5]. In contrast, generating iPGCs equivalent to e9.5–10.5 of development using ESC differentiation required 50 plates of hanging drop EBs, which takes 1 hour to set up from only two wells of undifferentiated ESCs. This yields on average 150,000–175,000 *Blimp1* positive iPGCs at day 6 of differentiation, resulting in more than 100-fold enrichment in cell numbers over embryonic dissections [40].

In conclusion, we propose that the ESC-to-PGC differentiation model is an essential tool for examining molecular events in PGC development. In this study we developed a model that specifically captures the *Blimp1*-positive stage of male PGC formation prior to the expression of Mvh protein. This period of germ cell development (prior to e11.5) is uniquely regulated in mammals and is not conserved with lower model organisms such as *Drosophila*, *C. elegans*, frog, and chick (for example, the role of *Blimp1*). Therefore, creating models that study the initial formation of mammalian PGCs such as the one described here, as well as extending this model to female ESC lines, will be critical to our understanding of the mechanisms that govern fundamental principles of inheritance via the germ line.

## Materials and Methods

### Ethics Statement

Mouse embryo dissection, breeding colony maintenance, and animal surgery were all performed following Institutional Approval for Appropriate Care and use of Laboratory animals by the UCLA Institutional Animal Care and Use Committee (Chancellor's Animal Research Committee (ARC)), Animal Welfare assurance number A3196-01.

### Cell Culture and EB Differentiation

All ESC lines in this study were maintained as described previously with lot-tested FBS (Hyclone Lot #ATJ33070) on inactivated CF-1 mouse embryonic fibroblasts (MEFs) [41]. Cells were passaged every three days at 5,300 cells/cm^2^. For EB formation, ESCs were subjected to MEF depletion by plating a single cell suspension on tissue culture dishes twice for five minutes each. Cells were seeded in drops of 20 microliters each containing 300 cells on the lids of Petri dishes with 5 mL PBS in the plate bottom and cultured in the absence of LIF for six days, with addition of 3.5 mL PBS on day 3 of differentiation. For ESC derivation, e3.5 blastocysts were isolated from homozygous *Blimp1^flox/flox^* (C57BL6/J) crosses and cultured in ESC media containing PD98059 (Cell Signaling) for four days. ESC lines were then passaged and maintained routinely. To generate *Blimp1* null ESCs, *Blimp1^flox/flox^* cells were transfected with pCAG-Cre:Gfp [42] and sorted to generate sublines.

### Mice

*Oct4-gfp* embryos were dissected and dissociated with TrypLE (Invitrogen) prior to flow cytometry or FACS. For teratoma analysis, 100,000 ESCs were injected into the testicles of SCID recipient mice and collected 6 weeks after transplant for histology [40].

### iPGC Colony Assay

iPGCs were sorted from EBs by FACS and re-plated on inactivated CF-1 MEFs. iPGCs were cultured in ESC media supplemented with 15 ngml^−1^ bFGF2 (R&D), 30 ngml^−1^ SCF (Peprotech), and 2 micromolar retinoic acid (Sigma), for five days as described previously [15]. Cells were cultured for five days with daily media changes followed by assaying for AP activity. EGCs were derived from iPGCs by culture of iPGCs for five days with LIF/SCF/bFGF2/RA, followed by passaging in LIF-only containing media for subsequent passages.

### Flow cytometry and FACS

Staining for SSEA1 (DSHB, 1∶200) and cKit (BD, 1∶200) was performed on ice. Indirect labeling was performed with Cy5-conjugated goat anti-mouse IgG and IgM (1∶500) and PE-conjugated goat anti-rat IgG (1∶1000) (Jackson ImmunoResearch). 7AAD or DAPI were added prior to all acquisitions to examine only live cells for downstream analyses with FlowJo software (TreeStar).

### Immunostaining

Embryoid bodies were fixed and embedded in paraffin according to standard protocols. For iPGC stains, cells were sorted by FACS and plated onto poly-lysine coated cover slips. The following antibodies were used at the indicated dilutions: SSEA1 (DSHB, 1∶100), Oct4 (1∶100, Santa Cruz), Mvh (1∶100, Abcam), and H3K27m3 (1∶500, Millipore). All samples were incubated with primary antibodies overnight at 4°C. Sections were washed, incubated with FITC anti-mouse IgM, TRITC anti-goat IgG, or FITC/TRITC conjugated anti-rabbit IgG antibodies (Jackson Immunoresearch) for 30 minutes at room temperature. Y chromosome FISH was performed on chromosome spreads. SSEA1 and Id4 immunohistochemical detection was performed using anti-Id4 (1∶100, Novus Biologicals) and anti-SSEA1 (DSHB) with standard protocols (Vector Labs).

### Real-time PCR

RNA was extracted from sorted samples using the RNEasy Micro Kit (Qiagen) and reverse-transcribed using Superscript RT II (Invitrogen). All gene expression analysis was performed using commercially available TaqMan Gene Expression Assays (Applied Biosystems), with the exception of *Id4*, which as examined by SYBR Green PCR (Roche). See Table S2 for additional primer information. CT values were normalized to *Gapdh* expression and expressed as fold change relative to a control cell type referenced in each experiment.

### Single-cell Real Time RT-PCR

Single cells were sorted by FACS and subjected to reverse transcription and specific target amplification of relevant genes using the Fluidigm BioMark 48.48 dynamic gene expression system according to manufacturer's instructions, with PCR performed by the UCLA Genotyping and Sequencing core facility. A dilution series of cells were used as detection controls and also to establish primer correlation coefficients and ensure linear amplification of amplicons. Heat map data was generated using Fluidigm Real Time PCR Analysis software.

### Microarray and Data Analysis

RNA extraction, labeling amplification and hybridization to Affymetrix Mouse Genome 430 2.0 arrays were performed as previously described [41]. Analysis was performed using model-based expression and invariant set probe normalization using D-Chip software [43]. Gene ontology (GO) terms were identified using DAVID [44], [45]. Microarray data is deposited under GEO accession number GSE33121.

### Bisulfite Sequencing

Genomic DNA was isolated from sorted samples (Zymo Research). Bisulfite conversion was performed using the EZ DNA Methylation Kit according to manufacturer's instructions (Zymo Research). PCR was performed on bisulfite converted genomic DNA and cloned into pCR2.1-TOPO (Invitrogen). Clones were sequenced and aligned using Lasergene software (DNASTAR). See Table S2 for PCR primer information.

### Southern Blot

*Prdm1/Blimp1* deletion was verified with dUTP-digoxigenin-labeled probe generated by PCR upstream of the deleted exons of *Blimp1* (fwd:5′- CTCGTGGCTCTTGTGTGTGT -3′, rev:5′- AACGCTGTACCCATGACTCC -3′), after digestion with *EcoRI*. Detection of wild type (15 kb), flox, (13.5 kb), and KO (10 kb) alleles of *Blimp1* have been described [46].

## Supporting Information

Figure S1 **Kinetics of EB formation and the transcriptional identity of iPGCs.** **A**: *Oct4-gfp* embryoid bodies at days 5–8 of differentiation. Scale bar = 500 microns. **B**: Quantification of EB cell viability recorded as the percent of 7AAD- cells at each time point by flow cytometry. **C**: Flow cytometry of the live cell EB fraction for Oct4-gfp and cKit at the corresponding time point. Blue oval indicates the Oct4-gfp+/cKit+ side population, which first appears at day 6. Oct4-gfp+/cKit^bright^ cells correspond to iPGCs. **D**: *Oct4-gfp* EBs at day 6 were stained with SSEA1 and cKit, and Oct4-gfp expression was examined in SSEA1+/cKit^bright^, SSEA1+/cKit^mid^ and SSEA1+/cKit^dim^ populations. **E**: PGC gene expression data for *Oct4-gfp* and J1-derived iPGCs and somatic cells. **F**: Somatic gene expression data for *Oct4-gfp* and J1-derived iPGCs and somatic cells.
(TIF)

Figure S2 ***Integrin Beta 3*** **is enriched in iPGCs.** Normalized signal intensity from probe sets for *Integrin beta 3 (Itgb3)* for ESCs and iPGCs were determined from the microarray presented in Figure 4A.
(TIF)

Table S1 DAVID Gene Ontology of transcriptional clusters identified between ESCs and iPGCs. Gene ontology analysis of genes corresponding to Affymetrix probe sets identified from each transcriptional cluster in Figure 4A differentially expressed more than 3-fold with p<0.01.
(DOC)

Table S2 Primers used in this study.
(DOCX)
