# Supplementary figures and images for: Single Cell Analysis Facilitates Staging of Blimp1-Dependent Primordial Germ Cells Derived from Mouse Embryonic Stem Cells
Source: PLoS One. 2011 Dec 15;6(12):e28960. doi: 10.1371/journal.pone.0028960 (PMC3240638; doi:10.1371/journal.pone.0028960)

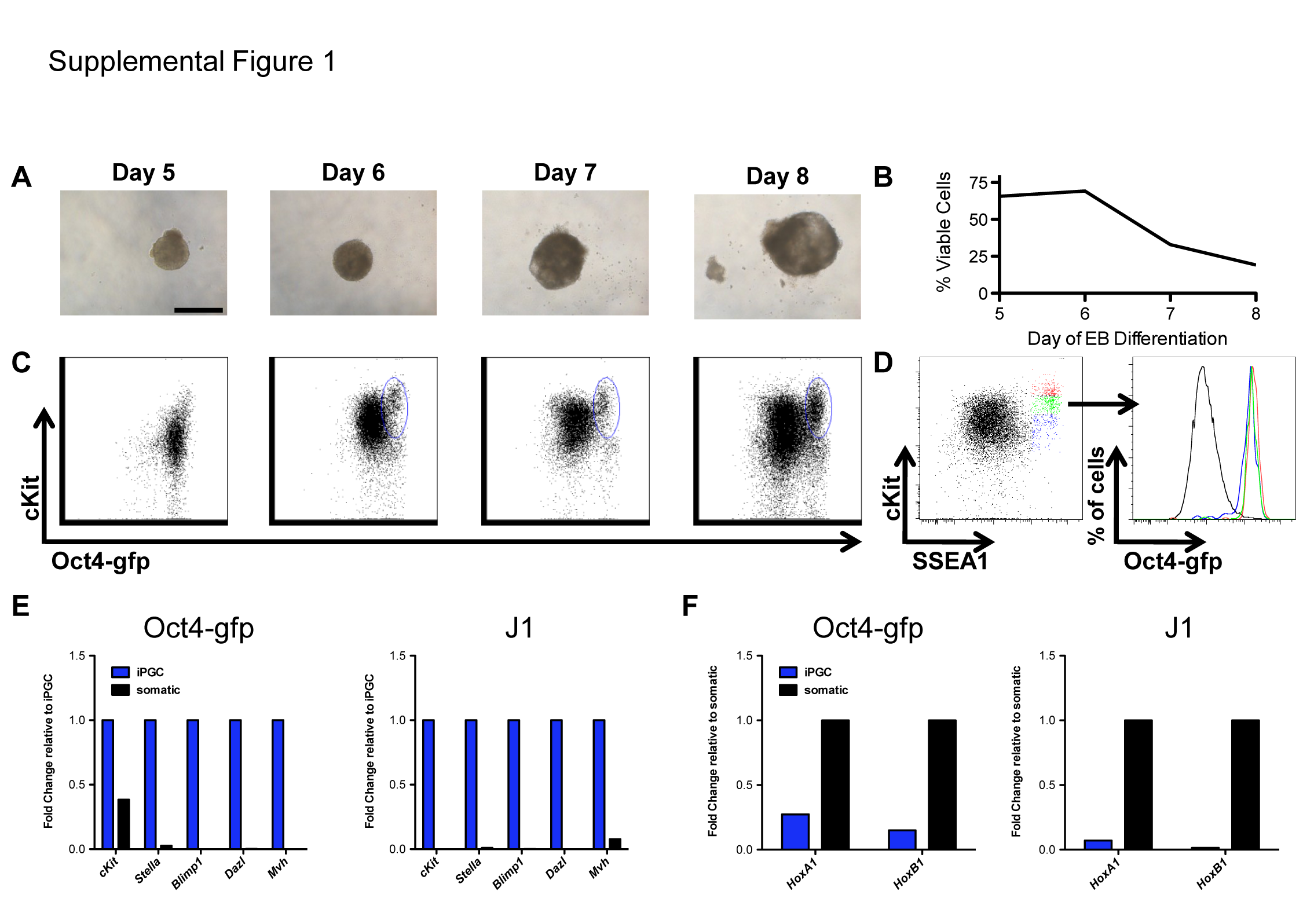

Supplement: Figure S1 — Kinetics of EB formation and the transcriptional identity of iPGCs. A: Oct4-gfp embryoid bodies at days 5–8 of differentiation. Scale bar = 500 microns. B: Quantification of EB cell viability recorded as the percent of 7AAD- cells at each time point by flow cytometry. C: Flow cytometry of the live cell EB fraction for Oct4-gfp and cKit at the corresponding time point. Blue oval indicates the Oct4-gfp+/cKit+ side population, which first appears at day 6. Oct4-gfp+/cKitbright cells correspond to iPGCs. D: Oct4-gfp EBs at day 6 were stained with SSEA1 and cKit, and Oct4-gfp expression was examined in SSEA1+/cKitbright, SSEA1+/cKitmid and SSEA1+/cKitdim populations. E: PGC gene expression data for Oct4-gfp and J1-derived iPGCs and somatic cells. F: Somatic gene expression data for Oct4-gfp and J1-derived iPGCs and somatic cells. (TIF) [file pone.0028960.s001.tif]

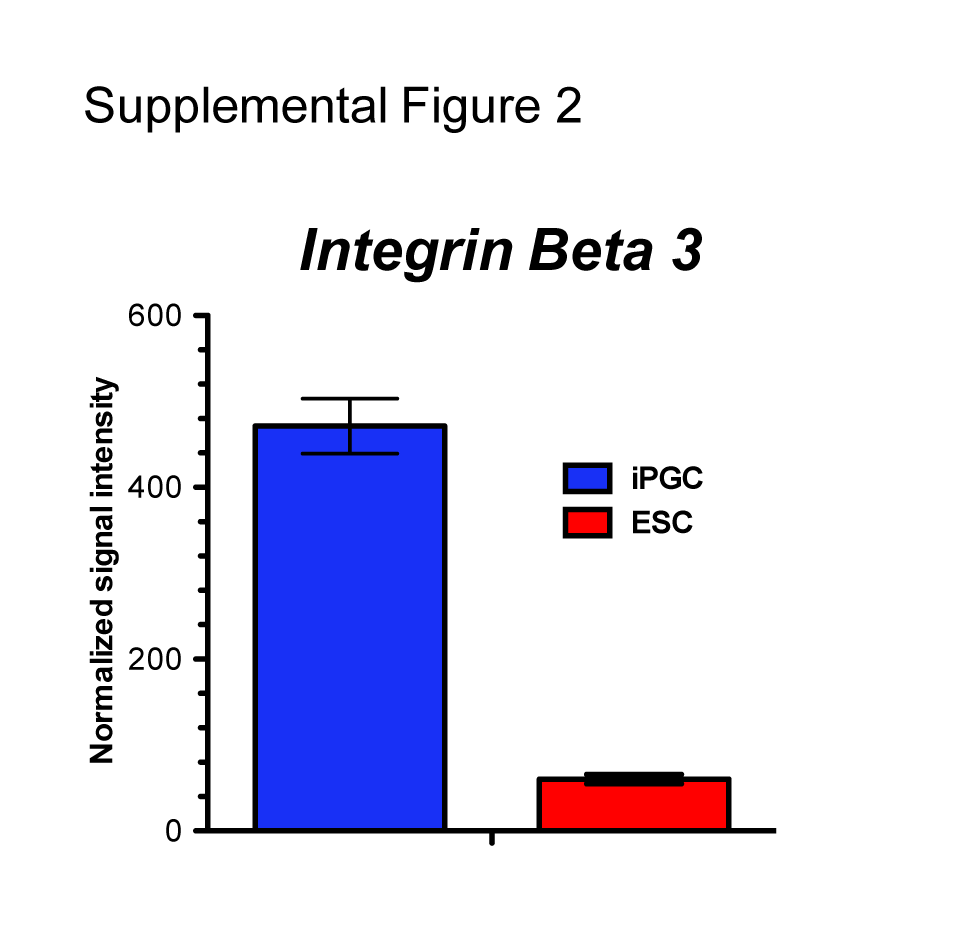

Supplement: Figure S2 — Integrin Beta 3 is enriched in iPGCs. Normalized signal intensity from probe sets for Integrin beta 3 (Itgb3) for ESCs and iPGCs were determined from the microarray presented in Figure 4A. (TIF) [file pone.0028960.s002.tif]
