# Supplementary material for: Single Cell Analysis Facilitates Staging of Blimp1-Dependent Primordial Germ Cells Derived from Mouse Embryonic Stem Cells
Source: PLoS One. 2011 Dec 15;6(12):e28960. doi: 10.1371/journal.pone.0028960 (PMC3240638; doi:10.1371/journal.pone.0028960)
Supplement: Table S1 — DAVID Gene Ontology of transcriptional clusters identified between ESCs and iPGCs. Gene ontology analysis of genes corresponding to Affymetrix probe sets identified from each transcriptional cluster in Figure 4A differentially expressed more than 3-fold with p<0.01. (DOC) [file pone.0028960.s003.doc]

**Supporting Information**

**Table S1. DAVID Gene Ontology of transcriptional clusters identified between ESCs and iPGCs.**

| **Cluster** | **Affymetrix probe sets** | **Top GO category** | **P-Value** | **Gene Examples** |
| --- | --- | --- | --- | --- |
| I | 126 | Transcription factor activity | 3.2E-05 | *Socs3, Sfrp1, Klf5, Klf4, Etv4, Klf9, Ets2, Hesx1, Hoxc13, Fgf17* |
| II | 126 | Cytoplasm | 8.0E-03 | *Rhox6, Rhox9, Tcl2, Dmrt3, Tex19.2, Map4k2, Id4* |
| III | 19 | Stress fiber | 2.5E-02 | *Fhl3, Anxa2, Parvb, Glipr2, Lmna, Itga5* |
| IV | 74 | Heart development | 4.90E-07 | *Tbx20, Cfc1, Fgf15, Msx1, Id2, Id3, Hand1, Wnt3, Wnt5b, Evx1,* |

Gene ontology analysis of genes corresponding to Affymetrix probe sets identified from each transcriptional cluster from Figure 4A differentially expressed more than than 3-fold with p<0.01.
