# Supplementary material for: Single Cell Analysis Facilitates Staging of Blimp1-Dependent Primordial Germ Cells Derived from Mouse Embryonic Stem Cells
Source: PLoS One. 2011 Dec 15;6(12):e28960. doi: 10.1371/journal.pone.0028960 (PMC3240638; doi:10.1371/journal.pone.0028960)
Supplement: Table S2 — Primers used in this study. (DOCX) [file pone.0028960.s004.docx]

**Supporting Information**

**Table S2. Primers used in this study.**

| **Gene** | **Primer** | **Sequence** |
| --- | --- | --- |
| Gapdh (RT) | FP | ACCACAGTCCATGCCATCAC |
|  | RP | TCCACCACCCTGTTGCTGTA |
| Id4 (RT) | FP | GAGACTCACCCTGCTTTGCT |
|  | RP | AGAATGCTGTCACCCTGCTT |
| Snrpn outer (BS) | FP | TATGTAATATGATATAGTTTAGAAATTAG |
|  | RP | AATAAACCCAAATCTAAAATATTTTAATC |
| Snrpn inner (BS) | FP | AATTTGTGTGATGTTTGTAATTATTTGG |
|  | RP | ATAAAATACACTTTCACTACTAAAATCC |
| IAP (BS) | FP | TTGTGTTTTAAGTGGTAAATAAATAATTTG |
|  | RP | CAAAAAAAACACACAAACCAAAAT |
| Xist outer (BS) | FP | TGGTTTGTTTAAGTAGAAGATATATTG |
|  | RP | AAAAATCTTACCAAAACATATCAAAAC |
| Xist inner (BS) | FP | GTATAGATAGGTGTGTGATTTAATG |
|  | RP | TTTAATATATTTTCTTAAATAAACC |

RT=RT-PCR, BS=bisulfite sequencing-PCR, FP=forward primer, RP=reverse primer
